# Ozonized Water in Microbial Control: Analysis of the Stability, In Vitro Biocidal Potential, and Cytotoxicity

**DOI:** 10.3390/biology10060525

**Published:** 2021-06-12

**Authors:** Laerte Marlon Conceição dos Santos, Eduardo Santos da Silva, Fabricia Oliveira Oliveira, Leticia de Alencar Pereira Rodrigues, Paulo Roberto Freitas Neves, Cássio Santana Meira, Greta Almeida Fernandes Moreira, Gabriela Monteiro Lobato, Carlos Nascimento, Marcelo Gerhardt, Arlene Souza Lessa, Luis Alberto Breda Mascarenhas, Bruna Aparecida Souza Machado

**Affiliations:** 1 University Center SENAI/CIMATEC, SENAI Institute of Innovation in Health Advanced Systems (ISI SAS), Salvador 41650-010, Bahia, Brazil; laerte.santos@fbter.org.br (L.M.C.d.S.); eduardossilva06@gmail.com (E.S.d.S.); fabricia.oliveira@fbter.org.br (F.O.O.); leticiap@fieb.org.br (L.d.A.P.R.); cassio.meira@fieb.org.br (C.S.M.); greta.moreira@fieb.org.br (G.A.F.M.); breda@fieb.org.br (L.A.B.M.); 2 University Center SENAI/CIMATEC, SENAI Computational Modeling and Industrial Technology, Salvador 41650-010, Bahia, Brazil; paulo.neves@fieb.org.br; 3 China Three Gorges Corporation—CTG Brazil, Rio Paraná Energia S.A. Rodovia MS-444 s/nº km 58, Ilha Solteira 79590-000, Selviria, Brazil; gabriela.lobato@ctgbr.com.br (G.M.L.); carlos.nascimento@ctgbr.com.br (C.N.); marcelo.gerhardt@ctgbr.com.br (M.G.); 4 Gonçalo Moniz Institute, FIOCRUZ Microscopy Service, Technological Platforms Network, Salvador 40296-710, Bahia, Brazil; arlene.lessa@fiocruz.br

**Keywords:** aqueous ozone, antimicrobial, antimicrobial resistance, sanitizer, cytotoxicity

## Abstract

**Simple Summary:**

Controlling microbial infections, especially nosocomial infection, is a task that continues to be a burden in many societies. The current pandemic has brought new concerns on this matter, mainly on how to better control the dissemination of microbial agents in the environment/surfaces and among humans. Therefore, the search for alternative methods and agents for disinfection is warranted. The aim of our study was to analyze the disinfecting potential of ozonized water in different in vitro tests. By performing microbiological and cell lineage in vitro assays, the biocidal effect of ozonized water was confirmed. Upon a short incubation time, bacterial strains and a yeast were killed by ozonized water, whereas there was no cytotoxicity in the mammalian cell line. These findings lead to the conclusion this agent can be safely tested in technologies for water spray disinfection devices.

**Abstract:**

O_3_ dissolved in water (or ozonized water) has been considered a potent antimicrobial agent, and this study aimed to test this through microbiological and in vitro assays. The stability of O_3_ was accessed following modifications of the physicochemical parameters of water, such as the temperature and pH, with or without buffering. Three concentrations of O_3_ (0.4, 0.6, and 0.8 ppm) dissolved in water were tested against different microorganisms, and an analysis of the cytotoxic effects was also conducted using the human ear fibroblast cell line (Hfib). Under the physicochemical conditions of 4 °C and pH 5, O_3_ remained the most stable and concentrated compared to pH 7 and water at 25 °C. Exposure to ozonized water resulted in high mortality rates for *Escherichia coli*, *Pseudomonas aeruginosa*, *Staphylococcus aureus*, *Enterococcus faecalis*, and *Candida albicans*. Scanning electron micrograph images indicate that the effects on osmotic stability due to cell wall lysis might be one of the killing mechanisms of ozonized water. The biocidal agent was biocompatible and presented no cytotoxic effect against Hfib cells. Therefore, due to its cytocompatibility and biocidal action, ozonized water can be considered a viable alternative for microbial control, being possible, for example, its use in disinfection processes.

## 1. Introduction

Ozone (O_3_) is a gas that has a natural configuration of three oxygen atoms. It is considered an elemental form of oxygen that occurs naturally in the Earth’s atmosphere, protecting the Earth from harmful solar ultraviolet radiation [1]. O_3_ is naturally produced by the irradiation of sunlight and the action of compounds, such as nitrogen oxides and volatile organic compounds [1]. O_3_ can also be produced artificially by the use of electricity generators [2,3], in which both air and oxygen can be used for gas formation. Its formation occurs from oxygen (O_2_) atoms that are supplied by the separation of the O_2_ molecules through the application of electrical discharges [2]. The free atom of O_2_ quickly combines with an available molecule of O_2_ (O + O_2_ > O_3_) forming O_3_ [2,4]. Once formed, the O_3_ rapidly decomposes into O_2_ (t1/2 = 20/30 min), and, for this reason, it must be produced in the place where it will be used [2,5].

As more antimicrobials are becoming ineffective to drug-resistant microorganisms, the focus should be shifted to alternative therapies. Research into new and non-antibiotic approaches to overcome infectious disease should be the focus of high priority research and development projects [6,7,8,9,10]. In addition, a search for methodologies aimed at reducing the spread and transmission rate for these pathogens, through contact either between humans or between humans and environments/surfaces, is warranted [11].

In several adapted in vitro sensitivity assays, the biocidal effect of O_3_ gas was confirmed, leading to a reduction in the load of bacteria, such as *Escherichia coli*, *Bacillus cereus*, *Pseudomonas aeruginosa*, *Staphylococcus aureus*, and *Bacillus cereus* (as well as *B. anthracis* and *B. subtilis*), among others [3,4,12,13,14]. This effect was also observed in species of fungi [15,16], such as the filamentous *Aspergillus brasiliensis* and the yeast *Candida albicans* [17]. Additionally, the antiviral activity of O_3_ was confirmed in Murine Norovirus (MNV-1), Bacteriophages, Hepatitis A Virus (HAV), and Polyvirus type 1 [18,19,20].

A problem associated with O_3_ gas is its toxicity [21,22]. One way to minimize this effect is its use dissolved in water. Given the toxicity of O_3_ and due to its reasonable solubility in water, increasing attention has been given to the use of this gas as a sanitizing agent [11,23]. O_3_ solubility in water indeed allows its immediate reaction with any soluble compounds and biomolecules present in biological fluids [23,24].

The generation of O_3_ in water (or other aqueous medium) occurs using O_3_ generators based on the dielectric barrier discharge method, also known as the corona effect [2]. This type of discharge is produced by applying a high voltage between two parallel electrodes, having between them a dielectric and a free space through which the air flows. Some systems perform this transformation from the ambient air, while others are coupled to high purity O_2_ cylinders. The air is bubbled directly into the water through an air outlet. After this ozonation process, it is necessary to perform the quantification and determination of the dissolved O_3_ concentration [25,26,27].

O_3_ dissolved in water (or ozonized water) can be defined as water obtained after an O_3_ infusion by the ozonation process. During this process, the only chemical reactions that can occur are those between O_3_ and inorganic matter, organic matter, or biological materials that are available, emphasizing that there is no reaction with the pure water itself [25,26]. Some parameters of evaluation and maintenance of the stability of ozonized water are important for this process of production and use, such as the pH and temperature, these being among other important factors for the half-life of the gas dissolved in water [26,28].

Among the advantages presented by ozonized water is the reduction of gas toxicity without the loss of the O_3_ sanitizing action. Experimental studies have proven the effectiveness of ozonized water as a microbicide agent. This feature is related to its powerful oxidizing action occurring from its decomposition process (O_(3)_ ⇋ O + O_(2)_), with the release of free radicals capable of acting in the inactivation of bacteria, fungi, viruses, and protozoans [12,15,16,19,27,29,30]. The biocidal action can also be identified by the direct form of action of this agent under the organic compounds of these microorganisms, affecting, in turn, their metabolisms [27,29].

Considering the need for new strategies for the control of microbial infections, the use of sanitizing agents, such as ozonized water, can be considered a safe strategy, even in emergencies, such as the current pandemic [11]. Therefore, the objective of this study was to evaluate the O_3_ stability in water under different pH and temperature conditions. In addition, the antimicrobial potential of ozonized water and its cytotoxic effects on mammalian cells were evaluated.

## 2. Materials and Methods

**Figure 1:**
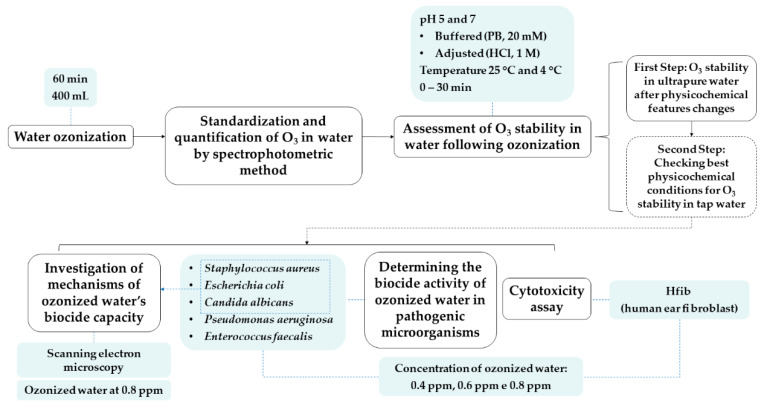
Experimental design. Synthesis of the methodology applied in this study.

Our experimental design is summarized in Figure 1. Details are given in the following sections.

### 2.1. Water Ozonation

O_3_ was dissolved in water using three jointly Ozonic C2 generators (Ozonic, Brazil, Indústria de Equipamentos de Ozônio Ltda, São Paulo, Brazil) for 60 min. This type of generator bubbles 2 g of O_3_ per hour, using ambient air as a feed gas captured through a 2 L/min compressor. The equipment displayed a flow rate of 0.9 L/min, and a rotameter was used for measurements. For all ozonation processes detailed herein, we used 400 mL of either ultrapure water (Merck, Darmstadt, Germany) or tap water. Either type of water was placed in a glass beaker covered with Parafilm^®^ M (Sigma-Aldrich, Saint Louis, MI, USA).

### 2.2. Standardization and Quantification of O_3_ in Water Using the Spectrophotometric Method

A spectrophotometric method for the quantification of aqueous O_3_ was standardized, based on a previously published method [31]. This iodometric method involves the oxidation of an iodine buffered solution and spectrophotometric measurements of the triiodide ion released by O_3_ [31]. Initially, the following solutions were prepared: (a) standard solution of potassium iodide (KI, 40 mM) andiodine (5 mM); and (b) reaction solution, containing potassium dihydrogen phosphate (KH_2_PO_4_, 100 mM), disodium hydrogen phosphate (Na_2_HPO_4_, 100 mM), and 2% KI. This reaction solution is used for the detection of high amounts of aqueous O_3_ [31].

Two calibration curves were prepared. In this case, the standard solution was diluted in the reaction solution producing concentrations from 0.015 to 8 ppm (parts per million). Each point was read immediately after the dilution of the standard solution at a wavelength absorbance of 352 nm (Spectrophotometer Model UV-M51 UV-Visible, BEL Photonics^®^, Monza, Italy). The assay was repeated for reproducibility purposes, but fewer concentration points were needed to achieve a valid standard curve. The absorbance values were then plotted using GraphPad Prism version 8.4.3 (686) for Windows (GraphPad Software, San Diego, CA, USA, www.graphpad.com, accessed on 25 March 2021). The curves were fitted in the software by choosing simple linear regression. As shown in Figure S1, the similarity of the calibration curves as well as the high R square indicate the validity of the generated curves and the reproducibility of the method.

For the detection of O_3_ in water, samples of ozonized water were incubated with the reaction solution (1:1) and allowed to react for 30 min. The incubation was performed in a dark chamber at 4 °C. The reaction solution was used as a blank. A Spectroquant^®^ Ozone Test kit 100607 (Merck, Darmstadt, Germany) was used to confirm the concentrations determined by the standardized method.

### 2.3. Assessment of O_3_ Stability in Water Following Ozonation

#### 2.3.1. First Step: O_3_ Stability in Ultrapure Water after Physicochemical Feature Changes

Considering the previous data on the stability of O_3_ in ultrapure water, which was previously considered the best type of water for increased O_3_ stability [26,32], changes were applied in its physicochemical parameters. Samples of ultrapure water with these different physicochemical characteristics are displayed in Table S1, and the changes are related to the temperature and pH. Regarding the temperature, water was kept in a fridge until it achieved a temperature of 4 °C, which was checked using a thermometer (K29-7070, Kasvi, Sao Jose dos Pinhais, PR, Brazil).

For pH-adjustment evaluations, hydrochloric acid (HCl) 1 M was used to adjust the pH, which was verified using a pH meter (Mettler Toledo FiveEasy™ Plus, Merck Darmstadt, Germany). Additionally, buffering was tested as a potential condition for stability by using sodium phosphate (PB) buffer (20 mM) with pH 5 or 7. The aim of these changes was to define which of these physicochemical parameters would maintain O_3_ longer in the water. After 1 h of ozonation for each test (Table S1) and considering the end of this process as the primary point of stability, aliquots of ozonized water were collected at 0, 5, 10, 15, 20, and 30 min, and determination of the concentration was performed as mentioned in the Section “Standardization and quantification of O_3_ in water using the spectrophotometric method”.

Considering 0 min as reference, the percentage of O_3_ reduction in water was calculated in all tested conditions (Table S1) and for each time of the stability curve. The calculation was performed using the formula % R = [(A − B)/A] ∗ 100, with A the amount in ppm of ozone dissolved in water at time 0 and B the amount of ozone dissolved in water after the study time. In the cases of certain tests, an ice bath was used to keep low temperatures in the glass beakers. The pH and temperature were checked before and after the ozonation processes. The experiment was in duplicate and repeated once. The data were then plotted using GraphPad Prism.

#### 2.3.2. Second Step: Checking Best Physicochemical Conditions for O_3_ Stability in Tap Water

Due to its easy obtainment and help in reducing costs during the development of ozonized water technologies, tests in tap water were also performed, applying the best physicochemical conditions found in the stability experiments using ultrapure water. Given the results with ultrapure water, only pH 5 (adjusted or buffered) was evaluated at different temperatures, which is shown in Table S2. The ozonizing and quantification practices were the same as those described above. However, considering that there was still detectable O_3_ in the tests with ultrapure water after 30 min, we added more points to the stability curve, with the following time points: 40, 50, and 60 min. The experiment was performed in duplicate and repeated once. The data were then plotted using GraphPad Prism.

### 2.4. Determining the Biocidal Activity of Ozonized Water in Pathogenic Microorganisms

The bacterial strains *Staphylococcus aureus* (ATCC 6538), *Escherichia coli* (ATCC 25922), *Pseudomonas aeruginosa* (ATCC 27853), and *Enterococcus faecalis* (ATCC 29212) were cultivated on tryptone soy agar (TSA) for 18–24 h at 37 °C. The yeast *Candida albicans* (ATCC 18804) was grown on Sabouraud chloramphenicol agar (SCA) for 48 h at 30 °C. After TSA or SCA growth, at least three well-isolated colonies were selected and transferred with an inoculation loop into a tube, which contained 5 mL of saline (0.85% NaCl). The turbidity of the broth culture was adjusted to obtain an optical turbidity comparable to that of the McFarland 0.5 standard solution, which contains approximately 1–2 × 10^8^ CFU mL^−1^ of *E. coli* ATCC 25922. Then, the microorganisms were diluted in saline to obtain concentrations of 5 × 10^5^, 5 × 10^4^, and 5 × 10^3^ CFU mL^−1^.

Based on previous studies and the unique characteristics of O_3_ reactions [33,34], the concentrations of 0.8, 0.6, and 0.4 ppm of O_3_ dissolved in tap water were tested in the assay, whose best water physicochemical features were used as follows: non-buffering, a temperature of 4 °C, and a pH 5 adjustment with HCl. Briefly, 1 mL of inoculum was incubated with 4.0 mL of ozonized water in the three concentrations of dissolved O_3_. The incubation time of these samples was 1 min at 20 °C. Following this interval, 0.5 mL of the inoculum, under the action of the different concentrations of O_3_ in the ozonized water, were added to 4.5 mL of a neutralizer (PB buffer pH 7, 20 mM, with 5 g/L sodium thiosulfate).

A control without exposure to ozonized water was performed for each dilution, although this control went through the same dilution process as the tests. Then, the samples were inoculated in duplicate in PCA (plate count agar) for bacteria and SCA for the yeast, which were grown at 37 and 30 °C for 24 and 48 h, respectively. The number of colonies on each agar plate was calculated after this incubation. Using the mean values of these counts, the death rate was calculated according to the following formula: Death rate (%) = (CFU of the control − CFU of the test)/CFU of the control) [13]. Additionally, the logarithmic scale (Log_10_) reduction factor was calculated using the formula RF = Log_10_ (A) − Log_10_ (B) (where A is the number of colonies recovered from the unexposed (control) and B is the number of colonies recovered from the exposed (test) to O_3_) [35]. The data were then plotted using GraphPad Prism.

### 2.5. Analysis of Microbials’ Morpho-Structural Changes Using Scanning Electron Microscopy

Scanning electron microscopy (SEM) was used to infer possible mechanisms of the killing capacity of ozonized tap water. In this case, to represent bacteria with distinctive cell walls and morphologies, one Gram-positive (*S. aureus*) and one Gram-negative bacteria (*E. coli*) were randomly included in this verification, as well as one yeast (*C. albicans*) of the present study. The turbidity of the broth culture was adjusted to obtain inoculums comparable to that of the McFarland 2 standard solution. The triplicated samples of the cells were centrifuged at 4000× *g*, 20 min, at room temperature.

The pellets were then resuspended in 100 µL of saline and incubated with 400 µL of ozonized water containing 0.8 ppm of O_3_. Following 1 min of incubation, a neutralizer (PB buffer pH 7, 20 mM with 5 g/L sodium thiosulfate) was used. A control without exposure to ozonized water was also performed and went through the same incubations and dilutions as the tests. Following another centrifugation, the microbial pellets were washed using saline and centrifuged again.

For SEM visualizations, the microorganisms in the pellets were fixed sequentially in two fixatives: (i) 2.5% glutaraldehyde in 0.1 M sodium cacodylate buffer, pH 7.4, for 1–2 h, followed by three washes with the same buffer; and (ii) 1% osmium tetroxide in 0.1 M sodium cacodylate for 1 h at room temperature, and washed three times with distilled water. Following fixation, they were then dehydrated in increasing concentrations of ethanol (30%, 50%, 70%, 90%, and absolute alcohol) and dried to the critical point using a drying machine with liquid CO_2_ as the transitional medium. The specimens were then examined by a JEOL, JSM-6390LV SEM.

### 2.6. Cytotoxicity Assay

The cell line Hfib (human ear fibroblast) was used for the cytotoxicity assays and obtained as previously stated [36]. The cells were cultivated in RPMI 1640 medium (Sigma-Aldrich, St. Louis, MO, USA) supplemented with 10% fetal bovine serum (FBS; GIBCO) and 50 µg mL^−1^ of gentamicin (Life, Carlsbad, CA, USA) at 37 °C and 5% of CO_2_. To confirm that the cell line was free of mycoplasma, this pathogenic microorganism was detected using the Mycoplasma Stain Kit (Sigma-Aldrich, St. Louis, MO, USA).

The cytotoxicity assay followed the method described by Colombo et al. [37] with certain modifications. Cells were plated in 96-well plates at 5 × 10^4^ cells mL^−1^ and incubated for 24 h at 37 °C and 5% CO_2_. Following that, the media was removed, and the cell lines were exposed to ozonized water (0.4, 0.6, and 0.8 ppm), using the best water physicochemical conditions (non-buffering, temperature of 4 °C, and pH 5 adjustment with HCl), for 1 min. After this incubation, the ozonized water was removed from the wells, which were washed with saline solution twice. Then, 20 µL of medium plus 10% AlamarBlue (Thermo Fisher Scientific, Carlsbad, CA, USA) were added in the wells. Incubations were followed for 4 h at 37 °C, and absorbance measurements were carried out at λ = 570 nm and λ = 600 nm.

The percentage of cell viability of exposed cells was calculated considering the absorbance of the control cells (without O_3_ exposure) as 100%. Then, using this value as a reference, we calculated the percentage for the exposed cells. Cell cultures containing 10% Triton X-100 were used as a positive control. The data were then plotted using GraphPad Prism. The same software was used for statistical analyses. The Kolmogorov–Smirnov test was used to verify the data distribution. Considering the non-parametric distribution, the Kruskal–Wallis test with Dunn’s post test was used to access statistical differences among the exposures. * represents *p* < 0.0001.

## 3. Results and discussion

### 3.1. O_3_ stability in Water

**Figure 2:**
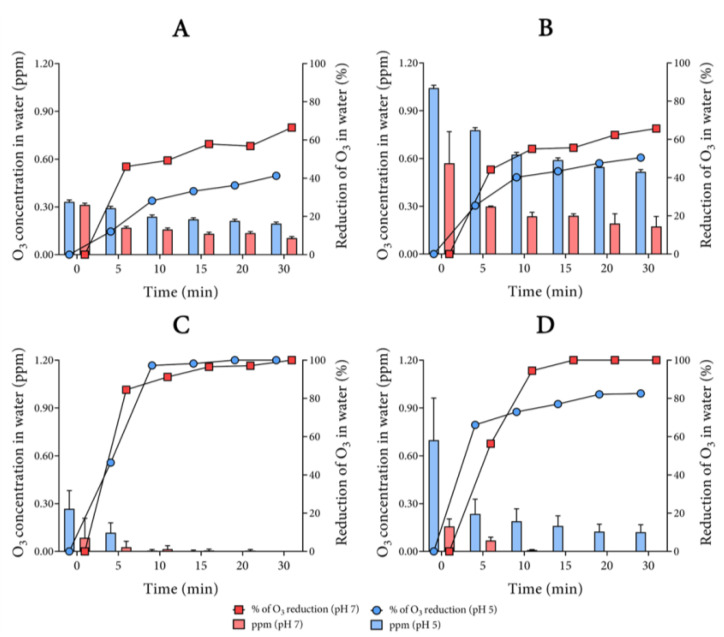
Ozone stability curves in ultrapure water. Ozonation of non-buffered ultrapure water at 25 °C (**A**) and 4 °C (**B**). The left y-axis is fitted the mean with standard deviation for the concentration in parts per million (ppm). The right y-axis is fitted the percentage of the O_3_ reduction in water. Both displayed y-axes were obtained from the same experiment. The percentage of O_3_ reduction in water was calculated considering the first sample (0 min) as the basis. Adjustment of pH was performed with 1 M HCl. Ozonation of buffered (PB, 20 mM) ultrapure water at 25 °C (**C**) and 4 °C (**D**).

Figure 2 details the decay of the concentration (left y-axis) and the percentage of O_3_ reduction (left y-axis) in ultrapure water for 30 min and under several physicochemical conditions (Table S1). In fact, the concentration of O_3_ in water decays depending on the physicochemical state of the water. From Conditions 1A and 2A (pH 5 and 7, respectively, at 25 °C) to Conditions 3A and 4A (pH 5 and 7, respectively, at 4 °C) (Table S1 and Figure 2A,B), O_3_ did not fall to 100%. The decrease in pH and temperature was clearly positive in terms of increasing the initial concentration of O_3_ in the water as well as decreasing the percentage of reduction. Specifically, when the temperature was decreased, the initial concentration increased from 0.571 ± 0.198 to 1.045 ± 0.021 ppm in ultrapure water with the pH adjusted to 7 and 4, respectively.

In addition, O_3_ took longer to decay at low temperatures, either at acidic or neutral pH. However, at pH 5, the concentration was always higher at any of the times on the stability curve (Figure 2B). We estimate that, at pH 5 and 4 °C, the concentration of approximately 0.3 ppm was maintained for about 30 min. This effect of pH on the slower decay of the O_3_ concentration in water was also observed in ozonized water at room temperature (Figure 2A), despite lower concentrations. The ultrapure water samples from Tests 2A (pH 7 at 25 °C) and 4A (pH 7 at 4 °C) (Table S1), initially adjusted to pH 7, displayed a reduction in pH at the end of bubbling, to 3.7 and 5, respectively.

Regarding buffering, the profile of the stability curves from Tests 5B and 6B (pH 5 and 7, respectively, at 25 °C) to Tests 7B and 8B (pH 5 and 7, respectively, at 4 °C) (Table S1) was not similar to the results with the samples with pH adjusted with HCl, but it was with regard to pH 5 at 4 °C (Figure 2C,D). However, the concentrations and percentages of reduction decreased and increased, respectively, in comparison with the same conditions in the adjusted pH of ultrapure water. We observed that, when using the 20 mM PB buffer, O_3_ was not detected after 5 min in Condition 6B (pH 7 at 25 °C) (Table S1 and Figure 2C). Despite this, we confirmed that cooling helped to increase the concentration and stability of O_3_ in ultrapure water, and this was more evident at pH 5.

At pH 5, after an initial drop after 5 min, the concentration of aqueous O_3_ remained virtually constant for 30 min (Figure 2D) in buffered ultrapure water, as it was for the water with pH adjusted with HCl (Figure 2B). In terms of concentration, while there was almost no increase in the concentration at pH 7 in both temperatures, in buffered water with pH 5, the concentration increased from 0.269 ± 0.112 to 0.695 ± 0.262 ppm at 25 and 4 °C, respectively. Comparing the four stability curves, buffering did not display effects on increasing the solubility of O_3_ in water.

This finding was confirmed at both temperatures; however, the difference in the stability curves at 25 °C was striking at both pH values with buffering inducing a complete decay of O_3_ in 10 min (Figure 2C), whereas adjustment of the pH with O_3_ was still seen in the water after 30 min (Figure 2A). This behavior was less drastic at 4 °C given that there was still an O_3_ presence in the buffered water at pH 5 (Figure 2D). In contrast, at pH 7, O_3_ was not detected after 10 min (Figure 2D). Considering that, under the adjustment conditions, there was a fluctuation of pH in ultrapure water, at either 25 or 4 °C (something that did not occur in buffered ultrapure waters), our findings indicate that non-buffering can lead to an increase in the O_3_ solubility in water.

**Figure 3:**
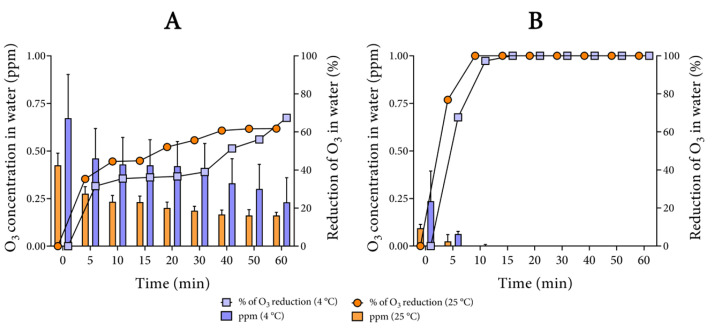
Sixty-minute ozone stability in tap water. Ozonation of non-buffered (**A**) and buffered (**B**) tap water. The left y-axis is fitted the mean with standard deviation for the concentration in parts per million (ppm). The right y-axis is fitted the percentage of the O_3_ reduction in water. Both displayed y-axes were obtained from the same experiment. The percentage of O_3_ reduction in water was calculated considering the first sample (0 min) as the basis. Adjustments of the pH and buffering were performed with 1 M HCl and 20 mM PB, respectively.

Bearing in mind that pH 5 appeared to be a key chemical parameter and the fact that tap water is known for its several organic compounds, which can reduce the O_3_ solubility and stability in water, we only tested this pH in two temperatures. Analyzing the results plotted in Figure 3, we noted that not only did a reduction in temperature increase the concentration of O_3_ in tap water (Figure 3A), but also the percentage of reduction in O_3_ in this water was slightly lower at 4 °C in comparison with 25 °C (Figure 3A). As with ultrapure water, the buffering clearly reduced the concentration and stability of O_3_ (Figure 3B). In comparison with ultrapure water, the buffering of tap water was more drastic as O_3_ was absent in the water after 5 min at either 25 or 4 °C.

**Figure 4:**
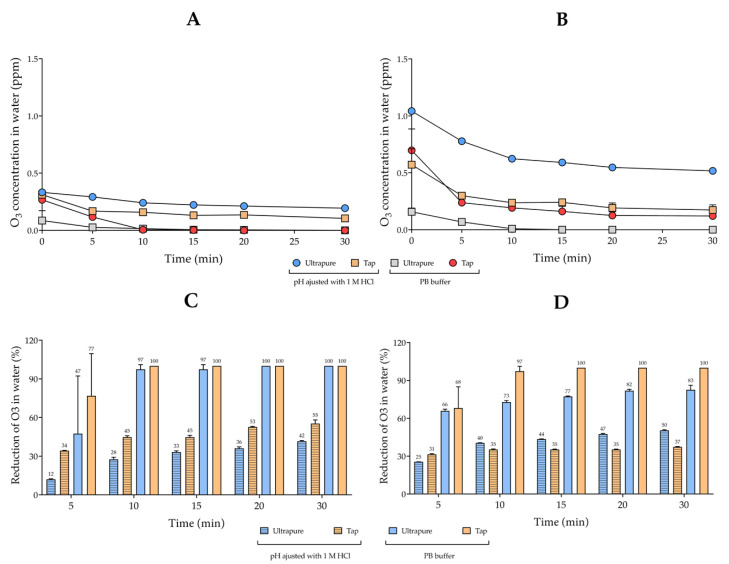
Comparisons of O_3_ concentration and stability in ultrapure and tap waters at pH 5. Ozonation of the two types of water at 25 °C (**A**) and 4 °C (**B**). The mean with standard deviation for the concentration in parts per million (ppm) were used to calculate the percentage of O_3_ reduction, considering the first sample (0 min) as the basis. The percentage values were then plotted as graphs for (**C**) 25 °C and (**D**) 4 °C. Adjustments of the pH and buffering were performed with 1 M HCl and 20 mM PB buffer, respectively.

When comparing the two conditions, at 4 °C, the pH-adjusted water maintained the O_3_ concentration between 0.65 and 0.25 ppm for 60 min, while, in the buffered water, the initial concentration of approximately 0.25 ppm decreased by about 70% in 5 min (Figure 3A and Figure 4B). At 25 °C, the outcomes were similar regarding both the percentage of reduction and concentration, but with lower values of concentration, which were almost not detected in the buffered samples (Figure 3A,B).

As shown in Figure 4A,B, when comparing the O_3_ concentrations of the two types of water in 30 min, it is apparent that the physicochemical parameters influenced the concentration of O_3_. The concentration of dissolved O_3_ was higher in ultrapure water after 1 h of ozonation, with initial concentrations of 1.045 ± 0.021 and 0.332 ± 0.011 ppm in water at temperatures of 4 and 25 °C, respectively. In contrast, the concentration of O_3_ in tap water remained lower than ultrapure water only at 4 °C, with initial concentrations of O_3_ of 0.673 ± 0.230 ppm (Figure 4A and Table S3). Comparing the O_3_ concentration in the two types of water with the addition of buffer solution, the ultrapure buffered water contained more dissolved O_3_ in relation to the buffered tap water. In numbers, at 4 °C, the initial concentrations were 0.695 ± 0.262 ppm for ultrapure and 0.237 ± 0.160 ppm for tap, while, at 25 °C, they were 0.269 ± 0.112 ppm for ultrapure and 0.094 ± 0.020 ppm for tap (Figure 4B and Table S3).

Figure 4C,D displays the comparative results of the percentage of O_3_ reduction in two types of water. O_3_ was more stable in tap water at 4 °C in comparison with ultrapure water at almost all time points of the stability curve, with the exception of 5 min. On the other hand, at 25 °C, the stability of O_3_ in ultrapure water was higher compared to tap water at all evaluated times in the stability curve. These outcomes indicate that lower temperatures were more important for stability than the purity of the water (Figure 4C and Table S4). The O_3_ reduction was clearly higher in buffered tap water, with 100% loss in 15 and 10 min at temperatures of 4 and 25 °C, respectively. The O_3_ reduction was also evident in the buffered ultrapure water, with a maximum reduction of 82.73% at 4 °C and 100% after 20 min in water at 25 °C (Figure 4D and Table S4).

These findings confirm that reducing the temperature and pH can help in O_3_ stability, even in the buffering condition that appeared to lack stability. Henry’s Law defines the solubility of O_3_, where a given temperature is linearly proportional to the partial pressure of the gas, when it is present in a solution. This means that the lower is the water temperature, the better is the dissolution of the gas in water [38]. At high temperatures, the rates of decomposition are higher, and, in addition, there is a reduction in the solubility of O_3_ in water [39].

The reaction of O_3_ with organic compounds in aqueous solution may occur through direct reaction, or indirectly involving reactions with hydroxyl radicals (OH^−^). This indirect reaction is fundamental to understand another important factor in the fast or slow decomposition of O_3_ in aqueous media: the pH levels. The stability of O_3_ in water decreases when the pH of the medium increases; when the pH is higher than 8.0, practically half of the introduced O_3_ is decomposed into various intermediate forms of oxygen in a period of 10 min [5,40].

No O_3_ was detected in buffers with pH 9.0 in previous studies, while a greater stability of O_3_ in solution occurred when the pH was 5.0 [24,41], as in the results demonstrated in this study. The concentration of OH^−^ can interfere considerably in the ozonation process as well as the acid–base balance of ozonized water, reflecting directly in the concentration of dissociated/non-dissociated forms present in the medium. However, the mathematical description is not yet well defined in relation to the performance of OH^−^ in the ozonation process and in the acid–base balance [26,32].

In relation to the presence of organic compounds in the water, a lower concentration (but not very significant) of O_3_ was observed in tap water. The presence of organic matter can cause a severe reduction of O_3_ in water [42,43,44], due to the rapid action of this agent with the aqueous compounds. However, it is precisely for this reason that O_3_ (in its gas form) is widely used in the treatment of wastewater [40,45,46]—for example, reacting with the dirtiness by the direct action of molecular O_3_ or by indirect reaction of the radical OH^−^ [32,46]. On the other hand, during comparisons with the two types of water, the stability of O_3_ in cold tap water was higher than that of the ultrapure water at the same temperature.

A possible explanation for this outcome is because the higher is the amount of solutes in water, the lower is the temperature changes, leading to a longer maintenance of the cold temperature [47,48] and, in turn, a slightly higher O_3_ stability in tap water. The presence of a solute in water makes the liquid state less organized because solutes or ions are freer to move at random. Therefore, the liquid water molecules possibly become more disordered in tap water, taking more energy to decrease this entropy as well as increasing the temperature [47,48,49]. Although we used an ice bath to keep the temperature as fixed as possible at 4 °C, during the ozonation process, some decreases occurred with values close to 0 °C. Taking this into consideration and the fact that physicochemical water parameters in Brazil are usually associated with the dispersion of a high amount of organic matter in the rivers, especially during rainfall season [50], ions (Ca^2+^, K^+^, and Na^+^) commonly encountered in soils are notorious for depressing the freezing point of water [48,49].

Another result, regarding the stability of O_3_, indicated that the buffering did not positively affect the increase of O_3_ solubility. This finding was confirmed at 25 °C; however, the difference in the stability curves at 4 °C was quite marked, especially at pH 5.0. Considering that, in the adjustment conditions, there was pH fluctuation (final pH of 4.0 after the experiments) in the water at either 25 or 4 °C—something that did not occur in buffered water—this is presented as another indication that non-buffering can lead to an increase of O_3_ solubility in the water due to pH fluctuations. If the oscillation is downwards, this can cause a higher permanence of O_3_ in the water, since the lower is the pH, the greater is the half-life of the gas in the water. A previous report indicated clearly that dissolved substances, either ionic or non-ionic, could affect the solubility of O_3_ in aqueous solutions, and buffering decreased both the solubility and stability of the gas [51].

### 3.2. Biocidal Effect of Ozonized Tap Water

**Figure 5:**
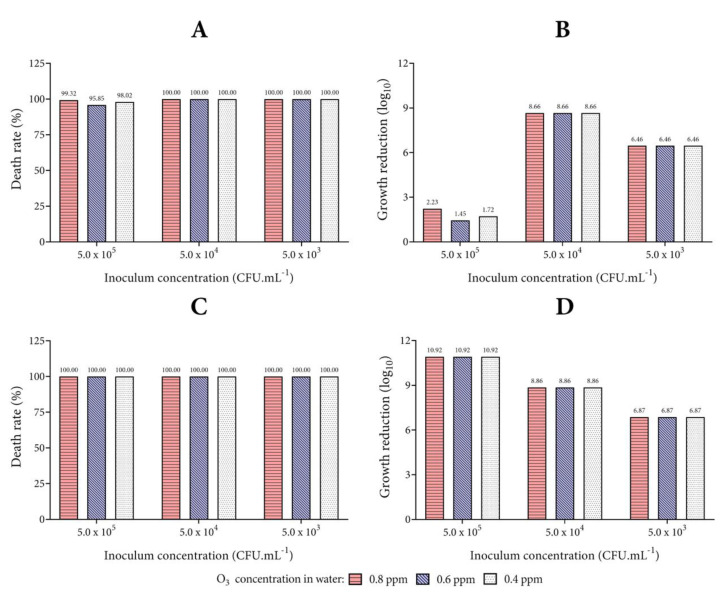
Action of ozonized water on Gram-negative bacteria. Analysis of the percentage of death and growth reduction in Log_10_ for the microorganisms *E. coli* (**A**,**B**) and *P. aeruginosa* (**C**,**D**). The concentrations of 0.8, 0.6, and 0.4 ppm of O_3_ dissolved in tap water were tested, and the number of colonies on each agar plate was calculated after incubation (1.0 mL of inoculum and 4.0 mL of ozonized water at 20 °C for 1 min).

**Figure 6:**
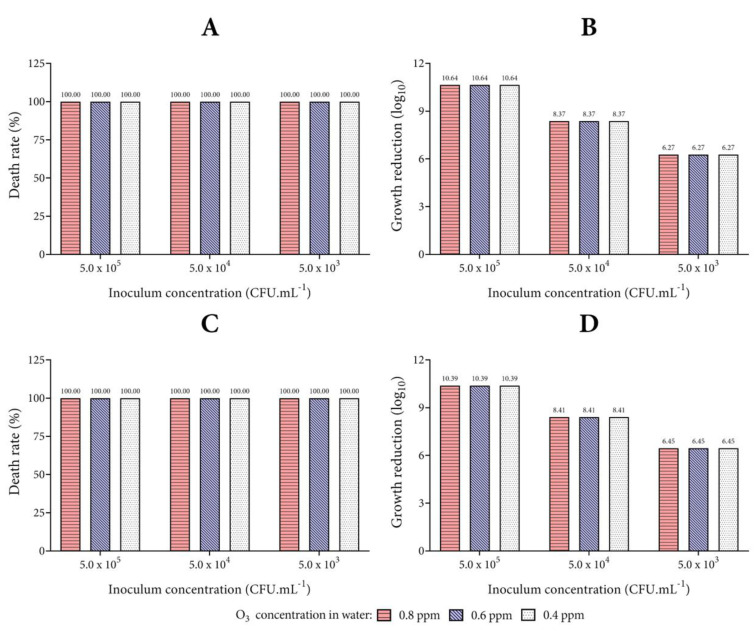
Action of ozonized water on Gram-positive bacteria. Analysis of the percentage of death and growth reduction in Log_10_ for the microorganisms *S. aureus* (**A**,**B**) and *E. faecalis* (**C**,**D**). The concentrations of 0.8, 0.6, and 0.4 ppm of O_3_ dissolved in tap water were tested, and the number of colonies on each agar plate was calculated after incubation (1.0 mL of inoculum and 4.0 mL of ozonized water at 20 °C for 1 min).

**Figure 7:**
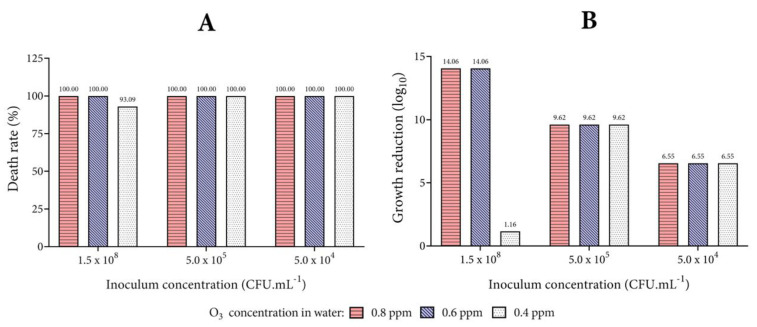
Action of ozonized water on yeast *C. albicans*. Analysis of the percentage of death (**A**) and growth reduction in Log_10_ (**B**) for *C. albicans*. The concentrations of 0.8, 0.6, and 0.4 ppm of O_3_ dissolved in tap water were tested, and the number of colonies on each agar plate was calculated after incubation (1.0 mL of inoculum and 4.0 mL of ozonized water at 20 °C for 1 min).

To evaluate the biocidal action of ozonized water (non-buffering, temperature of 4 °C, and pH 5 adjustment with HCl), the mortality rates of each microorganism studied are presented in Figure 5, Figure 6 and Figure 7. This condition of microbial death represents the effect of exposure of microorganisms for 1 min to ozonized water in different concentrations.

Figure 5 shows the results for the Gram-negative bacteria evaluated in the study. The exposure of *E. coli* to ozonized water showed a bacterial death rate higher than 95% from the inoculum concentration of 5 × 10^5^ CFU mL^−1^ in the three concentrations tested (0.4, 0.6, and 0.8 ppm). Better results in relation to *P. aeruginosa* were observed in this study. The mortality rate of the *P. aeruginosa* strain was equal to 100% from the lowest concentration of ozonized water (0.4 ppm) tested.

The average colony counts for each concentration of ozonized water were calculated and converted to Log_10_. When *E. coli* was exposed to ozonized water at the inoculum concentration of 5 × 10^5^ CFU mL^−1^, a reduction of >2.23 Log_10_ CFU mL^−1^ was observed. However, at an inoculum concentration equal to or lower than 5 × 10^4^ CFU mL^−1^, a higher reduction (>6 Log_10_ CFU mL^−1^) was observed. The logarithmic reduction for *P. aeruginosa* was higher than the results observed for *E. coli* after the same exposure time at different concentrations of ozonized water. 

While Figure 6 shows the results for the Gram-positive bacteria, Figure 7 displays the findings for the evaluated yeast *C. albicans*. Similar to the results demonstrated for the Gram-negative bacteria *P. aeruginosa*, the mortality rate of the *S. aureus* and *E. faecalis* strains were equal to 100% from the lowest concentration of ozonized water (0.4 ppm) tested. The best biocidal activity was observed against *C. albicans*, with a high death rate at the inoculum concentration of 1.5 × 10^8^ CFU mL^−1^. The logarithmic reduction for *S. aureus*, *E. faecalis*, and *C. albicans* proved to be higher than the results with *E. coli* in the same conditions tested. These results indicate the greater resistance of *E. coli* to treatment with ozonized water when in high inoculum concentrations.

Although *C. albicans* suffered growth reduction at the inoculum concentration of 1.5 × 10^8^ CFU mL^−1^, all the tested bacteria did not reduce growth as much at this concentration (for this reason, the inoculum concentration of 10^8^ for the bacteria was not plotted on the graphs), not allowing colony counting (bacterial confluence in the plaque). However, images of the plates (only bacteria) clearly revealed a microbial reduction following exposure to ozonized water in comparison with the control plates (Figures S3–S5). While in the test plates it was possible to notice isolated colonies, but still impossible to count them, the density of the biomass is clearly so high in the control plates that no isolated colonies can be observed (Figures S3–S5). On the other hand, *C. albicans* growth was strongly different from the bacteria evaluated in the present study (Figure S6). It is important to emphasize that the greater effect observed of the action of ozonized water in higher concentrations of microorganism inoculum can be explained because the higher is the inoculum, the greater is the colony forming unit in the control. For this reason, Log_10_ is higher at higher concentrations of microorganisms and is lower at lower concentrations. However, to better understand this, it is necessary to evaluate the results of Log_10_ and death rate together, noting that the reduction was mostly 100%, regardless of the inoculum used. The antimicrobial action of O_3_ herein showed is related to its powerful oxidizing action. O_3_ has been widely used as an antimicrobial, in both its gaseous and aqueous form, due to its wide range of microbiological, cleaning, and metabolic activities [6,13,15,16,24,34,45,52,53]. The antimicrobial action of O_3_ is related to its powerful oxidizing action. When it decomposes, this gas generates an oxidant reaction by the liberation of reactive oxygen species (ROS), including generating hydroxyl radicals (OH^−^) that have a greater oxidative potential (2.83 volts) over O_3_ and both act in the inactivation of several microorganisms [38,42,46,54,55].

In this study, the results show a considerable effectiveness of the ozonized water in the different concentrations of inoculums tested, except for the concentration of 1.5 × 10^8^ CFU mL^−1^ in most of the isolates. Megahed et al. [27] observed that the bacterial load is a strong predictor for the reduction of pathogens present in cattle manure exposed to ozonized water. In the study conducted by Białoszewski et al. [14], the concentration of O_3_ in water capable of eliminating the microorganisms contained in a suspension with a density of 1.5–5.0 × 10^8^ CFU mL^−1^ was up to nine times (1.2–3.6 ppm).

These findings were higher than the efficacy of the ozonized water of our study at the same density of microorganism tested. On the other hand, the use of ozonized water (0.4 and 8 ppm) against *E. coli* in an inoculum concentration equal to 3 × 10^4^ CFU mL^−1^ was efficient in decontaminating the hands [5]. However, it is important to consider that the number of medically important microorganisms, such as *S. aureus*, *P. mirabilis*, *Klebsiella* spp., and *E. coli*, present in intact areas of human skin can vary between 10^2^ and 10^6^ CFU/cm^2^ [56,57].

The susceptibility of microorganisms to O_3_ varies according to the physiological state of the cells, the pH of the medium, the temperature, the humidity, and the presence of additives, such as acids, surfactants, and sugars [3,58]. Relatively low concentrations of O_3_ and a short contact time were sufficient to inactivate pure suspensions of several microorganisms [44,58,59,60]. In this research, 1 min of exposure followed by the neutralization of O_3_ in the sodium thiosulfate buffer was effective in microbial reduction in the different concentrations of inoculum tested [13,58]. According to the structure of the cell wall, microorganisms can exhibit different sensitivities to ozonized water. In this study, we observed the greatest antimicrobial effect of ozonized water on Gram-positive bacteria [13].

These results are similar to those found by Zhang et al. [61] and Giuliani et al. [24], who, in their studies on the effect of ozonized water on different types of bacteria, reported that the effect of the ozonation process was greater in the action on Gram-positive microorganisms. However, in this work, a similar effect was observed for *P. aeruginosa*, which is a Gram-negative bacterium. Therefore, it is still necessary to investigate this behavior, taking into consideration the individual characteristics of each microorganism. Factors that can be associated to the distinct presentation of these results are the amount of lipids and lipoprotein present in the cell wall of the microorganisms, as well as the interaction between them and the behavior of the different membrane proteins [55,62].

Moore et al. [63] observed that, after ozonation (2 ppm per 4 h), Gram-negative bacteria (*E. coli*, *S. liquefaciens*, and *L. innocua*) were more sensitive to O_3_ compared with *S. aureus*. Białoszewsk et al. [14,33] concluded that all strains of *S. aureus*, *E. coli*, *E. hirae*, and *C. albicans* were rapidly killed in ozonized water with an O_3_ concentration in the range of 1.3–1.5 ppm during 1 min of exposure. *P. aeruginosa* ATCC 15442 was less sensitive to the effects of O_3_, with a decimal logarithm of reduction of 4.97 between the tested concentrations that was close to the limit level required by EN 1040:2006 [64]. The most evident action of ozonized water against *C. albicans* may be related to the mechanism of action of O_3_ in fungi through the oxidative mechanism in cell membranes.

The cell walls of fungi are multifaceted and composed of approximately 80% carbohydrates and 20% proteins and glycoproteins. The disulfide bonds in the fungal cell wall allow O_3_ to enter its cytoplasm and alter vital cell functions [65]. Similar results regarding the effectiveness of ozonized water under *C. albicans* could be observed in other studies. The ozonized water displayed inhibitory effects on the growth and colonization of *C. albicans* adhered to the surface of acrylic resins for a prosthesis base [66], and there was a reduction in the CFU count of *Candida* spp. in the saliva of patients with oral candidiasis [67].

Zargaran et al. [68] demonstrated that O_3_ was effective in controlling the growth of *C. albicans* and that it acted in the prevention of the formation of germ tubes and decreased the formation of biofilms. O_3_ acts under fungal species in a similar manner to the effect observed in viruses, promoting modifications to the genetic material [21,34,55,69], and one of the consequences is an increase in the resistance of some isolates to antifungals [68].

Considering the relevant biocidal aspect of ozone shown by our results, ozonized water is a potential sanitizer not only for decontamination of surfaces but as an agent to decrease the spreading of drug-resistant microorganisms in the environment. Some authors have previously stated the possibility of future pandemics, whose pathogenic agents may be bacteria for instance. Therefore, ozonized water can be useful in many spray devices currently available or under construction because it has high biocompatibility and could be used in these methodologies or devices to replace disinfectants considered toxic by regulatory agencies.

### 3.3. Microbials’ Morpho-Structural Changes Upon Incubation of Ozonized Tap Water

**Figure 8:**
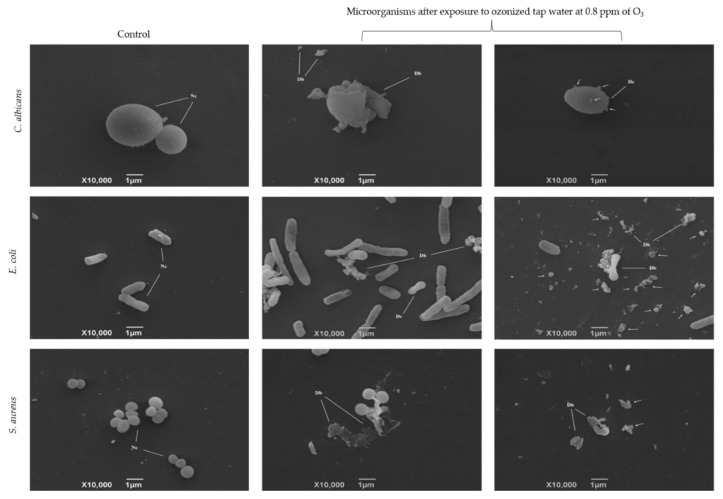
Scanning electron microscopy of *C. albicans*, *E. coli*, and *S. aureus* non-treated or treated with ozonized tap water at 0.8 ppm of O_3_. Microorganisms used in the test are displayed in left side of the chart. Conditions (control or test) for the assay are shown above. Normal cells (Nc), cell deformity (Dc), and cellular debris (Db) are indicated in the pictures. Arrows were added to the images to highlight other Db in the slides.

SEM micrograph visualizations were used to infer possible mechanisms for the observed decreases in the microorganisms’ growth. Figure 8 shows that, after exposure to ozonized tap water at 0.8 ppm of O_3_, morphological and structural changes were observed. For *C. albicans*, while the non-exposed cells did not display any damage or structural modifications (Figure 8), the incubation with ozonized water led to severe cell wall disruption as well as a high presence of cell debris (Figure 8).

Additionally, the formation of vesicles on *C. albicans*’s cell surface was noticed (Figure 8, arrows), indicating an increase in the permeability of the plasma membrane. In the case of *E. coli*, in comparing the control (Figure 8) with the exposed bacteria, we found severe cell dissipation (Figure 8), cell wall lysis, and the presence of cell debris (Figure 8, arrows). *S. aureus* suffered the same damages (Figure 8). These findings indicate that O_3_, even dissolved in water, not only directly induced damage in the cell walls and plasma membranes but also likely interfered in the cells’ osmolality.

Oxygen radicals cause cellular lysis by penetrating the microbial cell membrane in the presence of water, affecting its osmotic stability or interfering with cellular metabolism. However, additional biocidal mechanisms occur from the direct reaction of aqueous O_3_ with organic compounds of these microorganisms, thus influencing their metabolisms [17,27,30,70,71]. The SEM analyses performed in the present study indicate that ozonized tap water may have acted on the plasma membrane and cell walls of two of the bacteria and a yeast tested. The SEM micrographs demonstrated in the results corroborated this type of O_3_ mechanism of action, which previously inactivated several pathogenic microorganisms [72,73]. Such findings were observed by Thanomsub [72], where O_3_ inhibited the growth and caused structural changes, such as deformation and cell lysis in *E. coli*, *Salmonella* sp., *S. aureus*, and *Bacillus subtilis*, after 60 min of exposure. Clearly, the data found herein underscores the efficacy of O_3_ as an oxidizer, given the much shorter exposure time used and the achievement of structural damage in most of the *E. coli*, *S. aureus*, and *C. albicans* cells tested in the present research.

The formation of vesicles on the cell surface of *C. albicans* occurred due to the increased permeability of the plasma membrane and may represent the leakage of cellular constituents of cells during exposure to O_3_ [3,72,74]. However, it is important to note that cellular inactivation by O_3_ includes damage to the intracellular components, such as protein oxidation, damage to DNA, and alterations of enzymatic activity that cannot be observed from SEM [75]. Additionally, the presence of normal cells in the SEM images indicates that genes related to oxidative stress, such as SoxR, OxyR, and RpoS, favored the survival of those cells [54,76].

The rising resistance of microbes to the current sanitizers and pharmacological treatments raises concerns for future pandemics and control difficulties for the environmental and human dissemination of these pathogenic and resistant microorganisms. Therefore, the data presented herein indicate that the use of ozonized water in technologies for the disinfection of surfaces, medical instruments, and even humans may be an alternative and reliable approach to delay or stop the spread of microbes, as discussed by a recently published patent prospecting [11]. Ozone therapy has been indeed effective not only in dentistry but also in endocrinology and physical therapy [24,67,73,77,78,79,80,81], leading, in some cases, to the control of microbial infections while treating other illnesses [53,79,82]. Even methicillin-resistant *S. aureus* have been killed by O_3_ dissolved in oil during an experimental model of infected ulcer [53].

As another important example in this context, major world health organizations identified a highly pathogenic yeast *Candida auris*, which is one of the causes of nosocomial bloodstream infections, leading to outbreaks in several healthcare facilities around the globe, including Brazil, and associated with high mortality rates [83,84]. The control of such dangerous fungi has been difficult due its resistance toward most antifungals and the usual disinfectants used in hospital environments [85,86].

Although a recent study demonstrated *C. auris* resistance toward low concentrations and low exposure time of gaseous ozone [87], another study concluded a higher efficacy of repeated exposure of aqueous ozone, reducing the contamination of this yeast at a concentration range of 0.9–0.1 ppm [88], which is similar to the optimal concentration of 0.8 ppm in the present study. Considering this and the current efficacious use of dissolved ozone in many therapeutic and disinfecting procedures, ozonized tap water may be efficiently included in technologies for disinfection.

### 3.4. Cytotoxicity of Ozonized Tap Water

**Figure 9:**
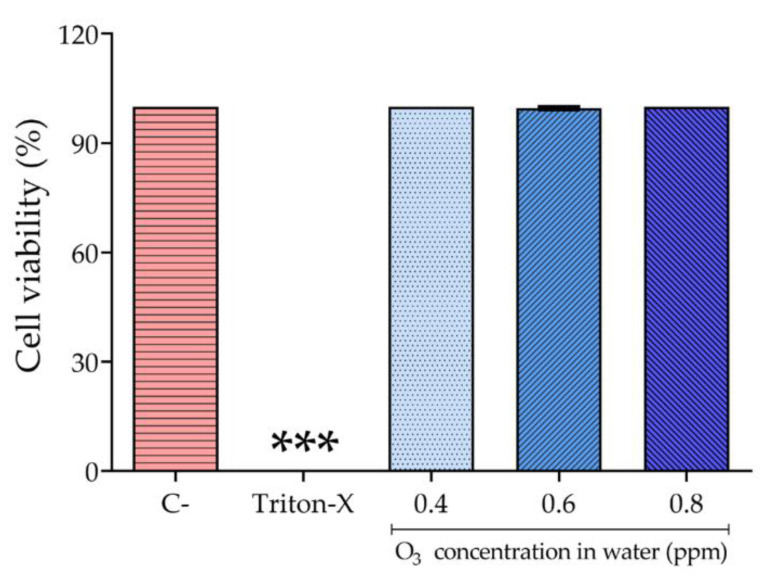
The behavior of Hfib cells upon ozonized water exposure. The different concentrations of ozonized water are displayed in the figure. C- represents the cells that were exposed to the medium only and 10% Triton-X was used as the positive control (cell death); *** Statistical difference between the positive control and the different concentrations of ozonated water.

Figure 9 shows that the mammalian cell line exposed to any concentration of ozonized water was as viable as non-exposed cells (C-), showing no degradation of cell membranes or release of intracellular content, when visualized in inverted optical microscopy. The exposed cells displayed 100% cell viability in almost all tested concentrations, with the exception of 0.6 ppm, in which one of the replicates achieved 98.02% viability. This outcome was reflected in the non-significance of Dunn’s statistical test with comparisons between exposure of the three concentrations and the non-exposed cells. On the other hand, the positive control (Triton-X), as expected, strongly affected viability, leading to complete death of the cells in a significant manner. These results indicate that 1 min exposure to ozonized water, for this cell line, did not present cytotoxic effects.

Before the application of ozonized water in human disinfection, it is important to verify the possibility of cytotoxic effects of ozonized water in mammalian cells. We tested this parameter and confirmed cytotoxic effects in this mammalian cell line. These findings are corroborated by previous studies, whose outcomes showed that some mammalian cells possess complex antioxidant systems, which do not allow ozonolysis at the cytoplasmic membrane and resist other oxidant properties of O_3_, even when the gas is dissolved in water or oils [37,77,89].

Not only antioxidant enzymes but also hydro-liposoluble compounds are considered components of the antioxidant systems of certain eukaryotic cells, such as blood cells and lineages cultured in vitro [77,78,89]. Additionally, it appears that O_3_ induces these cells to deliver other biochemical reactions in the mitochondrion. This organelle produces adenosine triphosphate (ATP) as a response to free radicals, and this energy molecule, in turn, improves the cell viability, given that it is used as a physiological improvement for the cells [37,77,89].

## 4. Conclusions

Given the results exposed, ozonized water is characterized as a potential antimicrobial agent. The O_3_ concentration increases according to the variation of the temperature and the pH value of the water. We demonstrated that the higher concentration of O_3_ in water at 4 °C and pH 5 resulted in greater efficacy in the decontamination process. In addition, ozonized water with a minimum concentration of 0.6 ppm was sufficient to clearly reduce the number of tested microorganisms, indicating that it can be used as a sanitizing agent. These results were corroborated with the SEM analysis (using ozonized water at 0.8 ppm of O_3_), which indicated that the effects on osmotic stability—due to cell wall lysis—might be one of the killing mechanisms of ozonized water. The ozonized water was also shown to be a biocompatible agent, as it did not present cytotoxic effects in mammalian cells. Therefore, due to its cytocompatibility and biocidal action, ozonized water was considered a viable alternative for microbial control, including for use in disinfection methodologies or devices (even for human use) to replace biocidal agents considered toxic by regulatory agencies.

## Data Availability

All the results found are available in this manuscript.

## Supplementary Materials

The following are available online at https://www.mdpi.com/article/10.3390/biology10060525/s1, Figure S1: Calibration curves for ozone quantification in water. The assay was performed two times with twenty-three (A) and ten (B) X values (iodide dilutions) for the detection of aqueous O_3_, respectively, Figure S2: Representative images of plating *Escherichia coli* exposed to different O_3_ concentrations. The concentrations of 0.8, 0.6, and 0.4 ppm of O_3_ dissolved in tap water were tested and the number of colonies of the agar plate duplicates was calculated after incubation (1.0 mL of inoculum and 4.0 mL of ozonized water at 20 °C for 1 min), Figure S3: Representative images of plating *Pseudomonas aeruginosa* exposed to different O_3_ concentrations. The concentrations of 0.8, 0.6, and 0.4 ppm of O_3_ dissolved in tap water were tested and the number of colonies of the agar plate duplicates was calculated after incubation (1.0 mL of inoculum and 4.0 mL of ozonized water at 20 °C for 1 min), Figure S4: Representative images of plating *Staphylococcus aureus* exposed to different O_3_ concentrations. The concentrations of 0.8, 0.6, and 0.4 ppm of O_3_ dissolved in tap water were tested and the number of colonies of the agar plate duplicates was calculated after incubation (1.0 mL of inoculum and 4.0 mL of ozonized water at 20 °C for 1 min), Figure S5: Representative images of plating *Enterococcus faecalis* exposed to different O_3_ concentrations. The concentrations of 0.8, 0.6, and 0.4 ppm of O_3_ dissolved in tap water were tested and the number of colonies of the agar plate duplicates was calculated after incubation (1.0 mL of inoculum and 4.0 mL of ozonized water at 20 °C for 1 min), Figure S6: Representative images of plating *Candida albicans* exposed to different O_3_ concentrations. The concentrations of 0.8, 0.6, and 0.4 ppm of O_3_ dissolved in tap water were tested and the number of colonies of the agar plate duplicates was calculated after incubation (1.0 mL of inoculum and 4.0 mL of ozonized water at 20 °C for 1 min), Table S1. Physicochemical changes in ultrapure water to assess ozone stability, Table S2. Physicochemical changes in tap water to assess ozone stability, Table S3. Differences among O_3_ concentrations in ultrapure and tap waters, Table S4. Comparisons of O_3_ stability in ultrapure and tap Waters.
