# Supplementary material for: Ozonized Water in Microbial Control: Analysis of the Stability, In Vitro Biocidal Potential, and Cytotoxicity
Source: Biology (Basel). 2021 Jun 12;10(6):525. doi: 10.3390/biology10060525 (PMC8231602; doi:10.3390/biology10060525)
Supplement: Supplementary file 1 [file biology-10-00525-s001.zip › biology-1229491-supplementary.pdf]

Supplementary Materials

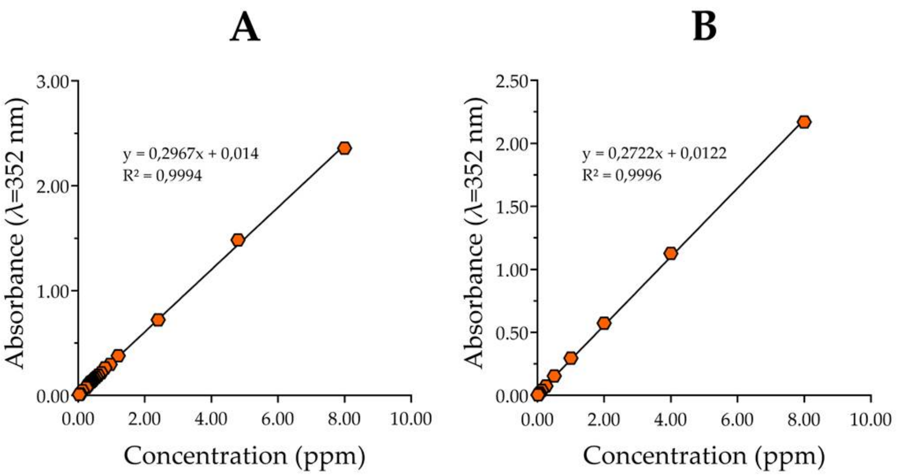

Figure S1. Calibration curves for ozone quantification in water. The assay was performed two times with twenty-three (A) and ten (B) X values (iodide dilutions) for the detection of aqueous O_3, respectively.

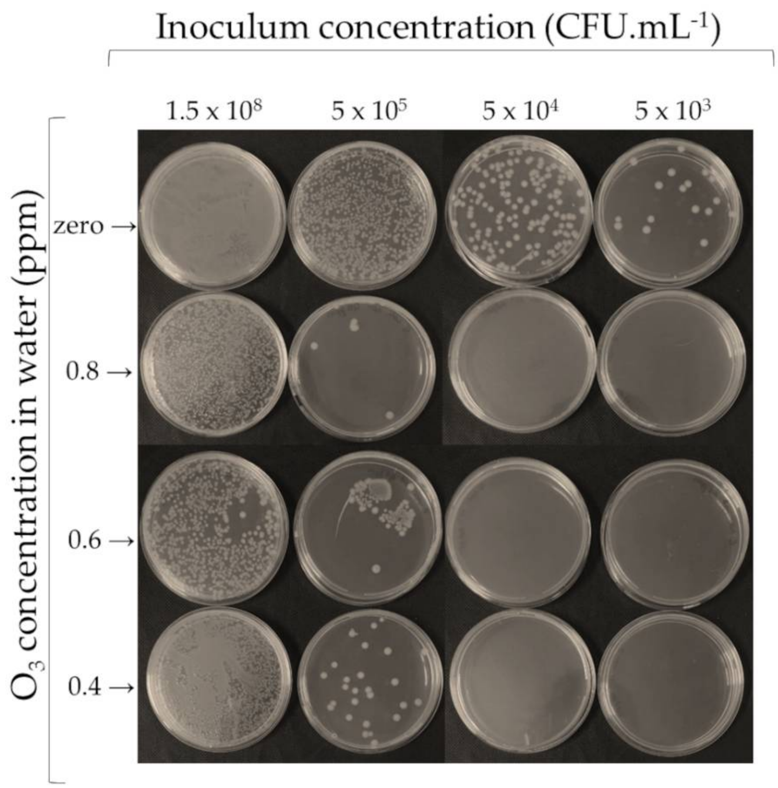

Figure S2. Representative images of plating *Escherichia coli* exposed to different O_3 concentrations. The concentrations of 0.8, 0.6, and 0.4 ppm of O_3 dissolved in tap water were tested and the number of colonies of the agar plate duplicates was calculated after incubation (1.0 mL of inoculum and 4.0 mL of ozonized water at 20 °C for 1 minute).

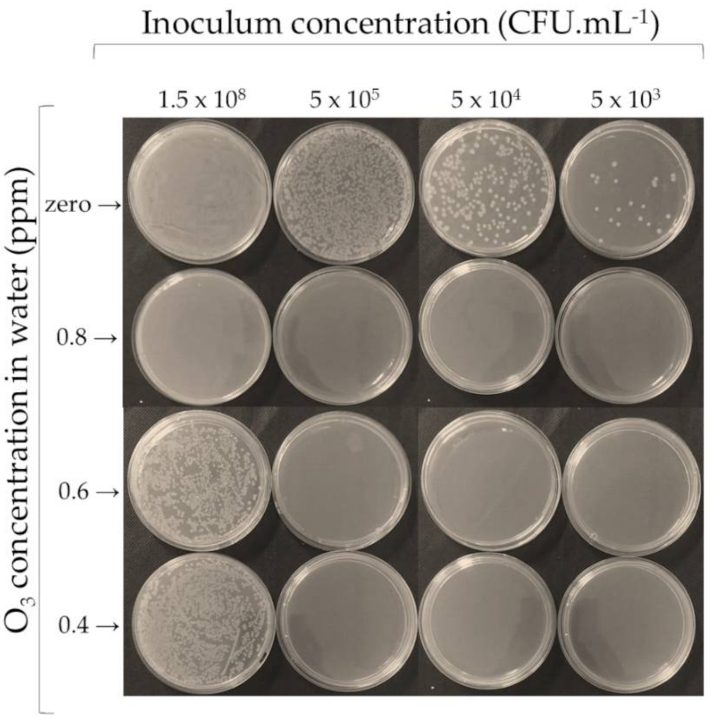

Figure S3. Representative images of plating *Pseudomonas aeruginosa* exposed to different O_3 concentrations. The concentrations of 0.8, 0.6, and 0.4 ppm of O_3 dissolved in tap water were tested and the number of colonies of the agar plate duplicates was calculated after incubation (1.0 mL of inoculum and 4.0 mL of ozonized water at 20 °C for 1 minute).

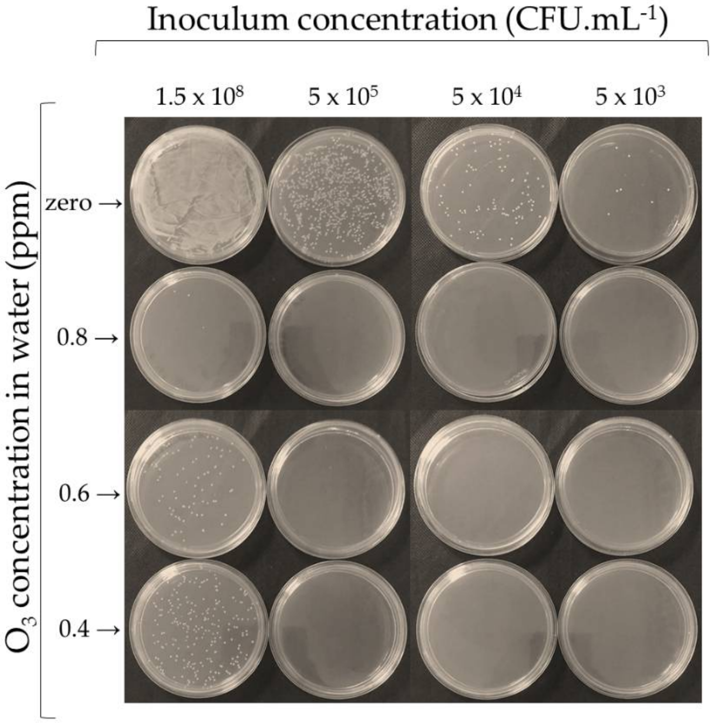

Figure S4. Representative images of plating *Staphylococcus aureus* exposed to different O_3 concentrations. The concentrations of 0.8, 0.6, and 0.4 ppm of O_3 dissolved in tap water were tested and the number of colonies of the agar plate duplicates was calculated after incubation (1.0 mL of inoculum and 4.0 mL of ozonized water at 20 °C for 1 minute).

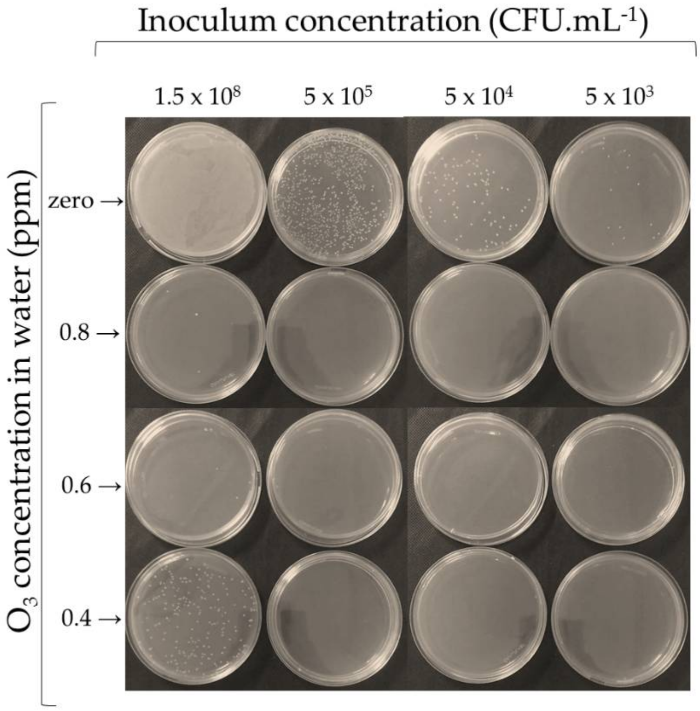

Figure S5. Representative images of plating *Enterococcus faecalis* exposed to different O_3 concentrations. The concentrations of 0.8, 0.6, and 0.4 ppm of O_3 dissolved in tap water were tested and the number of colonies of the agar plate duplicates was calculated after incubation (1.0 mL of inoculum and 4.0 mL of ozonized water at 20 °C for 1 minute).

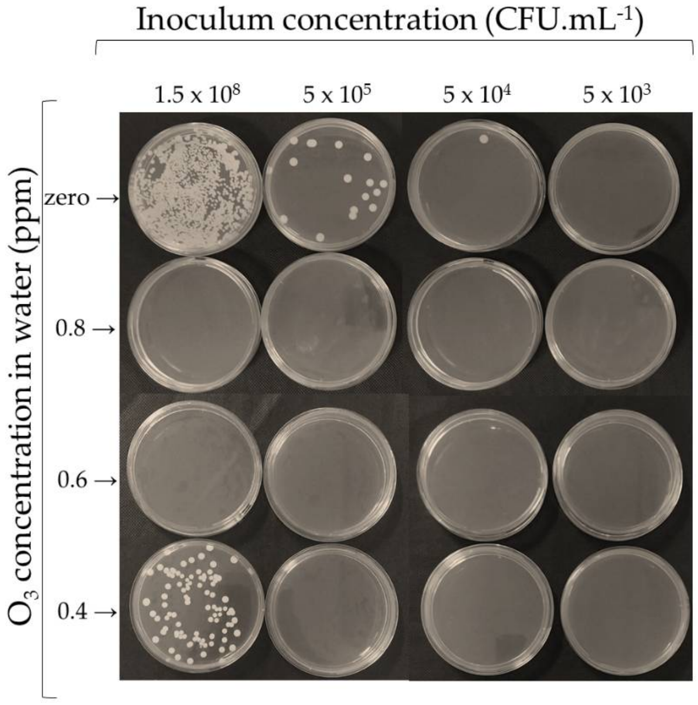

Figure S6. Representative images of plating *Candida albicans* exposed to different O_3 concentrations. The concentrations of 0.8, 0.6, and 0.4 ppm of O_3 dissolved in tap water were tested and the number of colonies of the agar plate duplicates was calculated after incubation (1.0 mL of inoculum and 4.0 mL of ozonized water at 20 °C for 1 minute).

Table S1. Physicochemical changes in ultrapure water to assess ozone stability.

Test identification	pH	Temperature (°C)
1A	5[#]	25
2A	7[#]	
3A	5[#]	4
4A	7[#]	
5B	5[*]	25
6B	7[*]	
7B	5[*]	4
8B	7[*]	

[#] Ajusted with HCl (1M). [*] Sodium phosphate buffer (20 mM)

Table S2. Physicochemical changes in ultrapure water to assess ozone stability.

Test identification	pH 5	Temperature (°C)
9C	Ajusted with HCl (1M)	25
10C	PB buffer (20 mM)	
11C	Ajusted with HCl (1M)	4
12C	PB buffer (20 mM)	

PB: sodium phosphate.

Table S3. Differences among O_3 concentrations in ultrapure and tap waters.

**Physicochemical condition of the water**		**Time**	**O_3 concentration in water (ppm)**	
**pH 5**	**Temperature (°C)**		**Ultrapure (Mean with SD*)**	**Tap (Mean with SD*)**
Ajusted with HCl (1M)	4	0	1.04 ± 0.02	0.67 ± 0.23
		5	0.78 ± 0.01	0.46 ± 0.16
		10	0.62 ± 0.01	0.43 ± 0.14
		15	0.59 ± 0.01	0.42 ± 0.13
		20	0.55 ± 0.01	0.42 ± 0.13
		30	0.52 ± 0.01	0.41 ± 0.13
	25	0	0.33 ± 0.01	0.42 ± 0.06
		5	0.29 ± 0.01	0.27 ± 0.04
		10	0.24 ± 0.01	0.23 ± 0.03
		15	0.22 ± 0.01	0.23 ± 0.03
		20	0.21 ± 0.01	0.20 ± 0.03
		30	0.19 ± 0.01	0.18 ± 0.02
PB buffer	4	0	0.69 ± 0.26	0.23 ± 0.16
		5	0.23 ± 0.09	0.06 ± 0.01
		10	0.18 ± 0.07	0.00 ± 0.01
		15	0.16 ± 0.06	0.00 ± 0.00
		20	0.12 ± 0.05	0.00 ± 0.00
		30	0.12 ± 0.04	0.00 ± 0.00
	25	0	0.27 ± 0.11	0.09 ± 0.02
		5	0.11 ± 0.06	0.02 ± 0.03
		10	0.01 ± 0.01	0.00 ± 0.00
		15	0.01 ± 0.01	0.00 ± 0.00
		20	0.00 ± 0.00	0.00 ± 0.00
		30	0.00 ± 0.00	0.00 ± 0.00

Mean and SD of two experiments. SD: standard deviation. Ppm: Parts per million. HCl: Hydrochloric acid. PB: sodium phosphate.

Table S4. Comparisons of O_3 stability in ultrapure and tap waters.

Physicochemical condition of the water		Time	Reduction of O_3 in water (%)*	
pH 5	Temperature (°C)		Ultrapure (Mean with SD)	Tap (Mean with SD)
Ajusted with HCl (1M)	4	5	25.50 ± 0.04	31.38 ± 0.53
		10	40.47 ± 0.14	35.29 ± 0.41
		15	43.50 ± 0.14	35.30 ± 0.30
		20	47.49 ± 0.45	35.30 ± 0.28
		30	50.36 ± 0.50	37.26 ± 0.36
	25	5	12.03 ± 0.38	34.21 ± 0.30
		10	37.58 ± 1.57	44.74 ± 1.05
		15	33.08 ± 1.03	44.74 ± 1.41
		20	36.09 ± 1.13	52.63 ± 0.42
		30	41.60 ± 0.59	55.26 ± 2.83
PB buffer	4	5	65.91 ± 1.29	68.11 ± 16.82
		10	72.82 ± 1.16	97.34 ± 3.77
		15	77.28 ± 0.39	100.00 ± 0.00
		20	81.82 ± 1.16	100.00 ± 0.00
		30	82.55 ± 3.61	100.00 ± 0.00
	25	5	47.45 ± 44.77	76.88 ± 32.70
		10	97.37 ± 3.72	100.00 ± 0.00
		15	97.37 ± 3.72	100.00 ± 0.00
		20	100.00 ± 0.00	100.00 ± 0.00
		30	100.00 ± 0.00	100.00 ± 0.00

*Percentage of reduction were calculated considering the concentration determined in the time zero of each experiment; Ppm: Parts per million; SD: standard deviation; HCl: Hydrochloric acid; PB: sodium phosphate.
